# Diagnostic Pitfalls and Therapeutic Strategies in the Treatment of Pancreatic Duct Haemorrhage

**DOI:** 10.1155/1997/96068

**Published:** 1997

**Authors:** P. J. Gallagher, G. Mclauchlin, P. C. Bornman, J. E. J. Krige, J. Thomson, I. N. Marks, J. Terblanche

**Affiliations:** 1 Surgical Gastroenterology, Groote Schuur Hospital University of Cape Town Observatory Cape Town 7925 South Africa; 2 Departments of Surgery and Medicine University of Cape Town Observatory Cape Town 7925 South Africa

## Abstract

Haemorrhage via the pancreatic duct, a rare cause of upper gastrointestinal bleeding (GIB), often poses a diagnostic dilemma. We analysed our experience with 10 patients (8 men, 2 women; mean age 44 years, range 34 – 62) treated during a 12 year period. All had a history of alcohol abuse and presented with major upper GIB requiring a median of 8 units (range 2 – 40) blood, transfusion. Nine had upper abdominal pain at the time of admission and nine had a history of pancreatitis. Upper gastroduodenal endoscopy (median 4; range 1 – 9), was diagnostic in only one. Side-viewing endoscopy showed bleeding from the pancreatic duct in 7 of 8 patients. Visceral aneurysms were demonstrated in 7 of 9 patients in whom coeliac angiography was carried out: (splenic artery 4, gastroduodenal artery 2, and pancreaticoduodenal artery 1). Two of 4 selective embolisations were successful. Six patients underwent distal pancreatectomy, 1 had gastroduodenal artery ligation and 1 died of coagulopathy following a total pancreatectomy. Pancreatic duct haemorrhage should be considered in patients with unexplained recurrent upper GIB, alcohol abuse and epigastric pain, particularly in those with established chronic pancreatitis. Selective angiography is essential for diagnosis and management. For bleeding sites in the head of the pancreas, embolisation should be attempted to avoid major resection. Distal pancreatectomy is preferred for splenic artery lesions.


*HPB Surgery*, 1997, Vol. 10, pp. 293–297
Reprints available directly from the publisher
Photocopying permitted by license only

© 1997 OPA (Overseas Publishers Association)
Amsterdam B.V. Published in The Netherlands
by Harwood Academic Publishers
Printed in India

### Diagnostic Pitfalls and Therapeutic Strategies in the Treatment of Pancreatic Duct Haemorrhage

P. J. GALLAGHER[a], G. McLAUCHLIN[a], P. C. BORNMAN[a], J. E. J. KRIGE[a], J. THOMSON[a], I. N. MARKS[b] and J. TERBLANCHE[a]

[a]*Surgical Gastroenterology, Groote Schuur Hospital and* [b]*Departments of Surgery and Medicine, University of Cape Town, Observatory 7925, Cape Town, South Africa*

*(Received 4 March 1996; In final form 25 October 1996)*

**Haemorrhage via the pancreatic duct, a rare cause of upper gastrointestinal bleeding (GIB), often poses a diagnostic dilemma. We analysed our experience with 10 patients (8 men, 2 women; mean age 44 years, range 34–62) treated during a 12 year period. All had a history of alcohol abuse and presented with major upper GIB requiring a median of 8 units (range 2–40) blood transfusion. Nine had upper abdominal pain at the time of admission and nine had a history of pancreatitis. Upper gastroduodenal endoscopy (median 4; range 1–9), was diagnostic in only one. Side-viewing endoscopy showed bleeding from the pancreatic duct in 7 of 8 patients. Visceral aneurysms were demonstrated in 7 of 9 patients in whom coeliac angiography was carried out: (splenic artery 4, gastroduodenal artery 2, and pancreatico-duodenal artery 1). Two of 4 selective embolisations were successful. Six patients underwent distal pancreatectomy, 1 had gastroduodenal artery ligation and 1 died of coagulopathy following a total pancreatectomy. Pancreatic duct haemorrhage should be considered in patients with unexplained recurrent upper GIB, alcohol abuse and epigastric pain, particularly in those with established chronic pancreatitis. Selective angiography is essential for diagnosis and management. For bleeding sites in the head of the pancreas, embolisation should be attempted to avoid major resection. Distal pancreatectomy is preferred for splenic artery lesions.**

*Keywords:* Gastrointestinal bleeding, chronic pancreatitis, pancreatic duct haemorrhage

### INTRODUCTION

Bleeding via the pancreatic duct is an uncommon but potentially lethal complication of chronic pancreatitis. In 1931 Lower and Farrell described the first case who at surgery was found to be bleeding into the pancreatic duct from a splenic artery aneurysm [1]. A further three cases were described by Degradi and Meister in 1959 [2]. No further reports were published until 1970 when the terms *haemosuccus pancreaticus* and *haemowirsungia* were introduced by Sandblom [3] and Bismuth *et al.* [4], respectively. The condition has also been designated *wirsungorrhagie* [5] and *haemoductal pancreatitis* [6, 7]. Despite this greater awareness and advances in diagnostic imaging techniques, the diagnosis and treatment are often delayed. Bleeding via the pancreatic duct is most

Correspondence: Professor: P. C. Bornman, E23 GIT Clinic, Groote Schuur Hospital, Observatory 7925, Cape Town South Africa. Telephone: 27-21-4043042, Facsimile: 27-21-4486461.

commonly related to alcohol-induced chronic pancreatitis but may also be the result of a variety of other pancreatic or peripancreatic conditions such as villous ductal tumours or atherosclerotic aneurysms [8]. This review analyses the management of pancreatic duct haemorrhage (PDH) emphasizing diagnostic pitfalls and therapeutic strategies.

### PATIENTS AND METHODS

Ten patients were treated in the Surgical Gastroenterology Unit with proven PDH during the 12 year period 1983 to 1994. Data were evaluated with regard to the mode of presentation, investigative findings including endoscopy, radiology, operative procedures, pathology and outcome.

There were 8 males and 2 females with a median age of 46 years (range 34–65). All patients had a history of chronic alcohol abuse and 9 had previous admissions for pancreatitis over a median period of 10 months (range 1–96) before presentation. Nine had severe epigastric pain associated with the bleeding. Eight had a median of 3 previous admissions (range 1–7) for self-limiting upper GIB for which no specific cause was found. All patients required initial resuscitation with a median of 6 units of blood (range 2–40). Two patients had previous surgery for upper GIB and in neither was a source of bleeding found. One underwent truncal vagotomy and pyloroplasty and a subsequent re-exploration for further bleeding and the other an exploratory laparotomy. All patients had an initial upper gastrointestinal endoscopy with an end-viewing scope.

Once the diagnosis was suspected a variable combination of abdominal computerised axial tomography (CT), ultrasound (US), side-viewing endoscopy and selective visceral angiography was performed.

### RESULTS

A median of 4 (range 1–9) upper gastrointestinal endoscopies using an end-viewing scope were performed for bleeding. In only 1 of these 35 endoscopies was bleeding from the ampulla identified. The CT and US revealed pancreatic pseudocysts and features of chronic pancreatitis in all 7 patients in whom these investigations were done and correlated accurately with the final anatomic bleeding site in 5 patients. In the 8 cases who had side-viewing endoscopy, blood was seen at the papilla in 5, in the duodenum in 1 and in 2 in whom there was some doubt, blood was aspirated from the pancreatic duct. Angiography revealed aneurysms in 7 of 9 patients (Fig. 1). In one of these cases the angiography was initially negative but revealed an aneurysm when the study was repeated during a bleeding episode. In the remaining two patients who had aneurysms in the tail, a cyst was demonstrated in the one case on CT and in the other the diagnosis was made at surgery.

Angiographic embolisation was attempted in 4 patients with aneurysms in the head of the pancreas. Embolisation was successful in 2 who had aneurysms of the pancreaticoduodenal

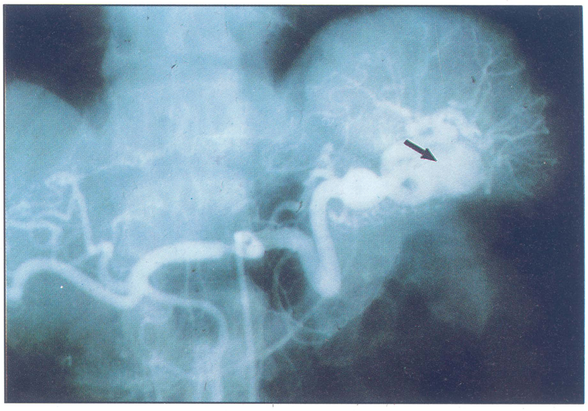

FIGURE 1 Selective coeliac angiography demonstrating a splenic aneurysm in the splenic hilum. (See Color Plate I).

artery and a side-branch of the splenic but failed in both cases where there was a large aneurysm of the gastroduodenal artery. Subsequently one of these cases underwent a total pancreatectomy and portal vein reconstruction and the other required ligation of the aneurysm through the cyst wall. The remaining 6 patients had a distal pancreatectomy and splenectomy.

There was one death as a result of blood loss secondary to a coagulopathy. In this patient there was a delay in diagnosis and embolisation failed. Bleeding associated with resection of the aneurysm and inflammatory mass in the head of the pancreas necessitated a total pancreatectomy and a vein graft replacement of the portal vein. Only one of the remaining 9 patients had morbidity – a common bile duct stricture and a pancreatic fistula following a distal pancreatectomy and splenectomy for which a choledochojejunostomy and a pancreaticojejunostomy was done.

A false aneurysm was confirmed histologically in 8 patients (operative specimen in 7 and biopsy in 1). In 4 patients a communication was demonstrated between the pancreatic duct and the false aneurysm (Fig. 2). There were no recurrences of PDH in the 9 survivors. Four were lost to follow-up after a median of 21 months (range 2–30) and 5 patients still attend the clinic after a median of 48 months (range 9–84).

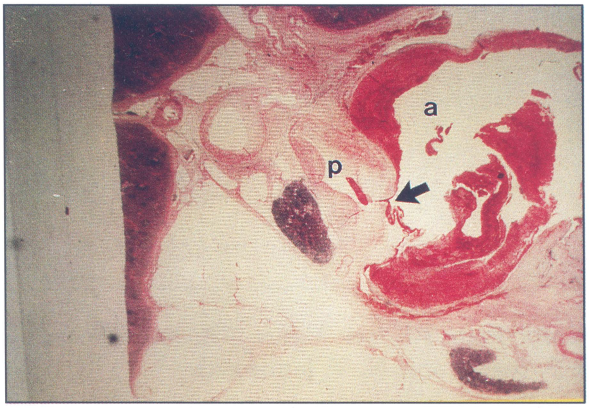

FIGURE 2 Histological section showing communication site (arrow) between the aneurysm (a) and pancreatic duct (b). (See Color Plate II).

### DISCUSSION

This study confirms that the commonest cause of PDH associated with alcohol induced chronic pancreatitis is a rupture of a peri- or intrapancreatic false aneurysm into the pancreatic duct. The pathogenesis is likely to be pseudocyst formation during an acute flare-up, with weakening of a contiguous vessel wall by activated pancreatic enzymes such as elastase and trypsin [9, 10]. The aneurysm then ruptures into the pseudocyst which is in communication with the pancreatic duct.

In this study causes of upper GIB that are common in alcoholic patients such as peptic ulcers, Mallory-Weiss tear, oesophageal and/or gastric varices and gastritis were initially presumed to be present prior to consideration of PDH. As in other series [7] there were often multiple endoscopies performed for self-limiting, recurrent upper GIB. Furthermore, the inadequacy of the end-viewing endoscope in leading to the diagnosis of PDH is confirmed.

One of the distinctive features of PDH is the associated abdominal pain caused by rapid duct distension due to blood in the duct. The clot formed may temporarily seal the vascular leak which would account for the self-limiting but recurrent nature of the bleeding [11]. The possibility that a clinically-acute attack of pancreatitis may precipitate PDH in the appropriate setting, however, cannot be excluded. This study confirms that a diagnostic tetrad exists for PDH consisting of a major upper GIB linked with pain, a negative upper GI endoscopy against a background of alcohol related pancreatitis. Under these circumstances PDH is the likely diagnosis if the side viewing endoscope reveals blood emanating from ampulla of Vater. If there is doubt as in two of our cases selective cannulation of the pancreatic duct with aspiration of blood is diagnostic. Pancreatography usually reveals changes consistent with chronic pancreatitis [12] and may point to the site of bleeding when a communicating pseudocyst is demonstrated.

In addition to confirming the diagnosis of chronic pancreatitis, the demonstration of cysts on CT and US is a useful additional marker of the site of bleeding [13, 14]. However, superior mesenteric and coeliac angiography is essential for accurate localisation of the involved vessels and allows an attempt at embolisation of the bleeding site. Angiography does not always demonstrate the source of bleeding between bleeding episodes and should be repeated when there is active bleeding to improve the diagnostic yield [11].

The splenic artery was the commonest source of bleeding and the gastroduodenal and pancreaticoduodenal artery or other branches were less frequently involved [7, 15–17]. When the head of the pancreas is involved angiographic embolisation may be successful in aneurysms involving smaller vessels [15–18]. In this series, however, embolisation was precluded in both cases with large gastroduodenal artery aneurysms in the head of the pancreas by their size and the presence of rich collateral vessels. Surgical resection of the pancreatic head remains the preferred definitive treatment. The type of resection [eg pylorus or duodenal preserving pancreatectomy] will be determined by the extent and severity of the inflammatory process. These are formidable procedures in high risk patients and has prompted some authors [15,18] to perform direct ligation of the aneurysm. Embolisation was not attempted in any case where the main splenic artery was involved and distal pancreatectomy and splenectomy remains the mainstay of treatment [11]. This can be performed with a low morbidity and mortality [15].

When features of the suggested tetrad are present in an upper GIB, an early side viewing endoscopy and a coeliac superior mesenteric artery angiogram must be performed urgently, preferably during the bleeding episode. Adoption of this policy avoids multiple admissions, transfusions, investigations and importantly inappropriate surgery. For aneurysms of smaller vessels, angiographic embolisation will be curative but large aneurysms in the head usually require surgery, preferably direct ligation to prevent a hazardous major pancreatic resection. Distal pancreatectomy is a safe and definitive operation for aneurysms of the splenic artery.

### *References*

[1] Lower, W. E. and Farrell, J. I. (1931). Aneurysm of the splenic artery: a report of a case and review of the literature, *Archives of Surgery*, **23**, 182–90.

[2] Degradi, A. E. and Meister, L. (1959). Massive upper gastrointestinal hemorrhage originating in the pancreas, *New England Journal of Medicine*, **36**, 269–72.

[3] Sandblom, P. (1970). Gastrointestinal hemorrhage through the pancreatic duct, *Annals of Surgery*, **171**, 61–66.

[4] Bismuth, H., Carton, B., Martin, E., Hernandez, C. and Hepp, J. (1970). Hemorragie digestive provenant des canaux pancreatiques (Hemowirsungies), *Archives Francaises des Maladies de l'appareil Digestif*, **59**, 735–41.

[5] Fraissinet, R., Sahal, J., Sarles, H. and Wirsungorrhagia (1978). Report of a case and review of the literature, *Gastroenterologie Clinique et Biologique*, **2**, 99–100.

[6] Longmire, W. P. and Rose, A. S. (1973). Haemoductal pancreatitis, *Surgical Gynecology & Obstetrics*, **136**, 246–50.

[7] Seiler, C. and Blumgart, L. H. (1993). Gastrointestinal haemorrhage due to splenic artery aneurysm pancreatic duct fistula in chronic pancreatitis, *HPB Surgery*, **7**, 149–55.

[8] Forsmark, C. E., Wilcox, C. M. and Gendell, J. H. (1992). Endoscopy negative upper gastrointestinal bleeding in a patient with chronic pancreatitis, *Gastroenterology*, **102**, 320–9.

[9] Stanley, J. C., Frey, C. F., Miller, T. A., Lindenauer, S. M. and Child, C. G. (1976). Major arterial haemorrhage. A complication of pancreatic pseudocysts and chronic pancreatitis, *Archives of Surgery*, **111**, 435–8.

[10] Goekas, M. C. (1968). The role of elastase in acute pancreatitis, *Archives of Pathology*, **86**, 112–41.

[11] Clay, R. P., Farnell, M. B., Lancaster, R., Weiland, L. H. and Gostout, C. J. (1985). Haemosuccus pancreaticus – an unusual cause of upper gastrointestinal bleeding, *Annals of Surgery*, **202**, 75–79

[12] Hall, R. I., Lavelle, M. I. and Venables, C. W. (1982). Chronic pancreatitis as a cause of gastrointestinal bleeding, *Gut*, **23**, 250–255.

[13] Hashimoto, B. E., Laing, F. C., Jeffery, R. B., Federle, M. P. (1984). Haemorrhagic fluid collections examined by ultrasound, *Radiology*, **150**, 805–808.

[14] Burke, J. W., Erickson, S. J., Kellum, C. D., Tegtmeyer, C. J., Williamson, B. R. J. and Hansen, M. F. (1986). Pseudo-aneurysms complicationg pancreatitis; detection by CT, *Radiology*, **161**, 447–450.

[15] Stabile, B. E., Wilson, S. E. and Debas, H. T. (1983). Reduced mortality from bleeding pseudocysts and pseudoaneurysms caused by pancreatitis, *Archives of Surgery*, **118**, 45–51.

[16] Frey, C. F. (1978). Pancreatic pseudocyst – Operative strategy, *Annals of Surgery*, **88**, 652–662.

[17] Huizinga, W. K. J., Kalideen, J. M., Bryer, J. V., Bell, P. S. H. and Baker, L. W. (1984). Control of major haemorrhage associated with pancreatic pseudocysts by transcatheter arterial embolization, *British Journal of Surgery*, **71**, 133–136.

[18] Yokoham, I., Hashimi, M. A., Stinivas, D., Shaikh, K. A., Levine, S. M., Sorokin, J. J. and Camishion, R. C. (1984). Wursungorrhagia or Haemoductal Pancreatitis: Reports of a case and review of the literature, *American Journal of Gastroenterology*, **79**, 764–768.

